# Sex-divergent effects of hindbrain GLP-1-producing neuron activation in rats

**DOI:** 10.3389/fnins.2023.1265080

**Published:** 2023-10-23

**Authors:** Lorena Lopez-Ferreras, Mohammed Asker, Jean-Philippe Krieger, Karolina Patrycja Skibicka

**Affiliations:** ^1^Department of Physiology/Metabolic Physiology, Institute of Neuroscience and Physiology, The Sahlgrenska Academy at the University of Gothenburg, Gothenburg, Sweden; ^2^Departamento de Biología Molecular, Instituto de Biomedicina y Departamento de Biología Molecular, Universidad de León, Spain; ^3^Institute of Veterinary Pharmacology and Toxicology, Vetsuisse, University of Zurich, Zurich, Switzerland; ^4^Department of Nutritional Sciences, Pennsylvania State University, University Park, PA, United States; ^5^Huck Institutes of the Life Sciences, Pennsylvania State University, University Park, PA, United States

**Keywords:** GLP-1, hindbrain, female rats, ingestive behavior, food-motivated behavior

## Abstract

Glucagon-like peptide-1 (GLP-1) analogs represent a new class of weight-loss medication, which has recently exponentially grown in popularity. GLP-1 is produced in the intestinal L cells in response to macronutrient intake, but it is also produced in the brain in a subset of neurons in the nucleus of the solitary tract (NTS). Exogenously-delivered GLP-1 analogs reduce food intake and food-motivated behavior in male and female rats, with some sex divergence of these effects in specific brain sites. These analogs potentially target GLP-1 receptors endogenously supplied by the gut and brain-produced GLP-1. The function of the NTS GLP-1-producing neurons [*Gcg* neurons] is still relatively unknown in rats. Moreover, even less is understood about the function of these neurons in females. We have recently developed a transgenic rat that expresses Cre under the *Gcg* promoter. Here, we interrogate this new animal model with optogenetics and chemogenetics to determine whether activation of the NTS GLP-1 neurons affects ingestive and motivated behavior in male and female rats. Optogenetic activation of the NTS *Gcg* neurons robustly reduced chow intake in both male and female rats. Interestingly, motivated behavior for a sucrose reward was reduced exclusively in females. To ensure that this unexpected sex difference was not activation method-specific, we next virally introduced excitatory DREADD receptors into the *Gcg* neurons and investigated the effect of chemogenetic activation of these neurons on ingestive and motivated behavior. Even upon chemogenetic activation, female rats reduced their motivation to obtain the sucrose reward, yet no effect on this behavior was observed in males. Our results show that activation of hindbrain *Gcg* neurons is sufficient to reduce food intake in both sexes. In females, but not males, *Gcg* neuron activation alone is also sufficient to reduce motivated behavior for sucrose. Thus, there is a sex difference in the ability of GLP-1-producing neuron activation to control motivated behavior for food.

## Introduction

Glucagon-like peptide-1 (GLP-1) is an incretin postprandially released from the intestinal L cells. The analogs of GLP-1 have been a successful treatment for type 2 diabetes and are also recently emerging as a blockbuster anti-obesity drug. With the soaring use of GLP-1-related formulations, it is increasingly urgent to understand the full physiological function of the GLP-1 system. The gut is not the only source of GLP-1 in the body; GLP-1 can also be produced in the hindbrain, primarily in the caudal nucleus of the solitary tract (cNTS) in rodents and humans (Turton et al., 1996; Merchenthaler et al., 1999; Llewellyn-Smith et al., 2011; Richards et al., 2014; Zheng et al., 2015a). The function of these hindbrain neurons is of great interest since GLP-1-based therapeutics have direct access to central GLP-1R and likely reduce eating via central GLP-1 receptors (GLP-1R) (Kanoski et al., 2016; Knudsen et al., 2016). The latter are endogenously activated by the ligands produced by the cNTS GLP-1-producing neurons.

Several previous studies examined the function of *Gcg* neurons, largely in mice. In these reports, chemogenetic stimulation of these GLP-1-producing neurons was shown to reduce food intake in fed and fasted states in lean or diet-obese mice, while body weight was only altered in obese mice (Gaykema et al., 2017). Activation of these neurons also improves glucose homeostasis (Shi et al., 2017). However, this previous literature has either studied exclusively male mice or did not report sex used (Gaykema et al., 2017; Liu et al., 2017; Shi et al., 2017) or data were not disaggregated or analyzed by sex (Holt et al., 2019); leaving the role of these neurons in females largely unexplored. Considering that women make up at least half of the patients receiving GLP-1-based therapeutics, the question of the role of these neurons and the receptors they innervate in females is of clinical relevance.

GLP-1 is a post-translational cleavage product of the *Gcg*-encoded preproglucagon protein. We have recently generated a transgenic *Gcg*-*Cre* rat model that expresses iCre under the control of the *Gcg* promoter (Zheng et al., 2022). This model was extensively characterized to show Cre expression as expected in the hindbrain, intestine, and pancreas (Zheng et al., 2022). Chemogenetic activation of hindbrain GLP-1-producing neurons in this rat model reduced chow intake, and this reduction was GLP-1R-dependent (Zheng et al., 2022). Interestingly, when these rats were offered a choice of chow or a more palatable food, peanut butter, chemogenetic GLP-1-producing neuron activation reduced exclusively chow intake in males but only peanut butter intake in females. These results potentially hint at, but do not directly test, a sex difference in the role of hindbrain GLP-1-producing neurons in the control of food reward behavior. A wealth of literature indicates that brain GLP-1R activation results in reduced food reward or motivated behavior for food (Dickson et al., 2012; Mietlicki-Baase et al., 2013; Skibicka, 2013; Alhadeff et al., 2014; Richard et al., 2015; Vogel et al., 2016; Lopez-Ferreras et al., 2018; Maske et al., 2018; Terrill et al., 2019). While much of that literature also focuses on male rodents, we have previously shown that substantial sex differences exist specifically in how GLP-1R activation affects food-motivated behavior (Richard et al., 2016; Lopez-Ferreras et al., 2019). For example, we found that female rats display a significantly less robust suppression of food-motivated behavior after pharmacological activation of GLP-1R in the lateral hypothalamus and the supramammillary nucleus compared to male rats (Lopez-Ferreras et al., 2018, 2019).

In the current study, we set out to ask whether there is a sex divergence in the role of GLP-1-producing neuron activation in food reward behavior by evaluating the effect of hindbrain GLP-1-producing neuron activation on sucrose-motivated behavior in male and female rats. To ensure our results are not restricted to a specific activation method, both optogenetic and chemogenetic GLP-1-producing neuron activation were tested.

## Materials and methods

### Animals

All work involving rats was approved by the Institutional Animal Care and Use Committee of the Sahlgrenska Academy at the University of Gothenburg, Sweden and was performed in adherence with the Guide for the Care and Use of Laboratory Animals. Unless otherwise noted, adult rats were individually housed in plastic cages upon arrival at the animal facility, with wood-chip bedding in a vivarium with a 12:12 h light:dark cycle and *ad libitum* access to rodent chow and water.

*Gcg*-*Cre* knock-in rats were generated as we have recently described (Zheng et al., 2022). Briefly, *iCre* was inserted after the final coding sequence of exon 6 before the 3′ UTR of the *Gcg* gene, using previously described CRISPR/Cas9 technology. Heterozygous (Het) *Gcg*-*Cre* male rats were shipped from China to the Janvier facility in France to rederive the line. Rats were genotyped on arrival, and biopsies were stored. *Gcg*-Cre males were naturally mated with Janvier SD females. The resulting embryos were then transferred to pseudopregnant SD females. The offspring genotype was determined from tail biopsies by Transnetyx, Inc. (Cordova, TN) using real-time polymerase chain reaction. Wild type (WT) and Het progeny born at Janvier were shipped to the University of Gothenburg. Heterozygous, and not homozygous, progeny was used since we found that homozygous (+/+) *Gcg*-Cre rats from our Janvier colony had reduced *Gcg* mRNA expression, and females also displayed increased body weight (as expected for a *Gcg* knockdown), while Het rats showed no significant changes in *Gcg* expression or body weight (Zheng et al., 2022).

### Operant conditioning

The operant conditioning procedure was used to assess the motivation to obtain a food reward in the form of a 45-mg sucrose pellet. Training and testing were conducted in the early to mid-light cycle in rat conditioning chambers (Med-Associates, Georgia, VT, USA) as we previously described (Dickson et al., 2012; Lopez-Ferreras et al., 2018). Training was conducted in four stages in *ad libitum*-fed rats. Rats were first trained on the fixed ratio 1 (FR1) schedule in 30-min sessions (a single press on the active lever results in the delivery of one 45 mg sucrose pellet), followed by FR3 and FR5 (3 and 5 presses required per pellet, respectively), where a minimum of 30 responses per 30-min session on the active lever is required for advancement to the next schedule. Finally, the rats were trained in progressive ratio [PR (Hodos, 1961)] until stable responding was achieved (~7 sessions), where the cost of a reward was progressively increased for each following reward in order to determine the amount of work the rat is willing to put in to obtain the reward. Responding was considered stable when the number of pellets earned per session did not differ more than 15% between three consecutive sessions. All operant response testing was performed after the responses stabilized under the PR schedule in 60-min sessions. The number of rewards earned and lever presses made to earn the rewards were measured. Food seeking, defined as the number of head pokes into the food dispenser, was also measured. Additionally, horizontal locomotor activity was measured inside the operant boxes with infrared beams for the entire 60-min period of testing.

### Optogenetic stimulation of cNTS *Gcg* neurons

Viral injections and implantation of ferrule-capped optical fibers (200 μm core, NA 0.37 for optogenetic stimulation; Doric Lenses, Canada) were performed as we previously described (Eerola et al., 2022). For somatic stimulation of GLP-1-producing neurons, Het *Gcg*-*Cre* rats (males: *N* = 5; females: *N* = 6) were bilaterally injected with an AAV encoding Cre-dependent ChR2 [AAV5-eSYN-DIO-hChR2 (E123T/T156A)-EGFP (VB2125)] at the following coordinates for caudal NTS: at the skull ridge; ±0.8 from the midline; and −7.9 mm ventral from the skull. The virus was infused bilaterally at 0.5 μl per side, at 0.1 μl/min. An 8-mm optical fiber was stereotaxically placed over cNTS to reach the same coordinates as above, 5 weeks after viral infusions. Fibers were secured to the skull with bone screws and dental cement. For both procedures, rats were anesthetized with ketamine and placed into a stereotaxic device (Stoelting).

### *In vivo* photostimulation

Photostimulation was performed as previously described (Eerola et al., 2022). All optogenetic stimulation experimental hardware was purchased from Doric Lenses, Canada. Fiber optic cannulas were connected to a two-channel LED driver (LEDRVP_2CH_1000) with a patchcord (MFP_200/220/900–0.53_1M_FC-ZF1.25(F)) via a fiber optic rotary joint (LEDFRJ). The strength of the output 470 nm blue light from the LED driver was optimized to 10 mW/mm^2^ for each fiber optic cannula (80–90 mA) using a photodiode power sensor (PM100D/S121C, Thorlabs Inc., Newton, USA). For the stimulation of hindbrain *Gcg* neurons, a 470-nm light was delivered for 10 ms pulses at 20 Hz for 1 s, repeated every 4 s using the Doric Hybrid MultiLED Driver control software. The stimulation commenced 5 min prior to the PR session and continued 30 min into the session. Prior to the motivated behavior test, rats received 50% of their normal overnight food ration to increase the baseline motivated behavior against which the *Gcg* neuron optoactivation effect was tested. 30 min after termination of the stimulation, rats were transferred to their home cage and offered pre-weighed amounts of chow. Cumulative chow intake was measured manually 1 and 24 h after the food was offered. Rat body weights were assessed before and after the 24-h food intake measurement. The photostimulation test was repeated 48 h later using a within-subjects crossover design, such that each rat was stimulated (or received sham stimulation) in a counterbalanced fashion. Correct AAV targeting of the cNTS was confirmed at the termination of the behavioral experiments, where cannula tracks and Cre-mediated AAV reporter expression were confirmed using a fluorescence microscope.

### Chemogenetic stimulation of cNTS *Gcg* neurons

Introduction of DREADD: AAV2-hSyn-DIO-hM3D(Gq)-mCherry (Addgene 44361) was delivered bilaterally into the cNTS (0.5 μl; 0.1 μl/min) through a cannula implanted with the tip positioned at the following coordinates: at the level of the occipital suture, ±0.7 mm from midline; 5.9 mm ventral from skull of adult male and female *Gcg*-Cre Het rats (Hets: *N* = 10 males, 10 females; WT controls: *N* = 10 males, 7 females). The injector extended 2.0 mm beyond the cannula tip. Correct AAV targeting of the cNTS was confirmed at the termination of the behavioral experiments, where cannula tracks and Cre-mediated AAV reporter expression was confirmed using a fluorescence microscope.

Food-motivated behavior measurement: Rats were unilaterally injected through the cNTS cannula with CNO [0.5 μg in 0.3 μl of 1% DMSO in an artificial cerebrospinal fluid (ACSF) vehicle] or vehicle alone. Prior to the motivated behavior test, rats received 50% of their normal overnight food ration to increase the baseline motivated behavior against which the *Gcg* neuron activation effect was tested. Rats were placed into the testing chambers 10 min after CNO injections.

## Results

### Anorexic effect of NTS *Gcg* neuron activation

**Figure 1:**
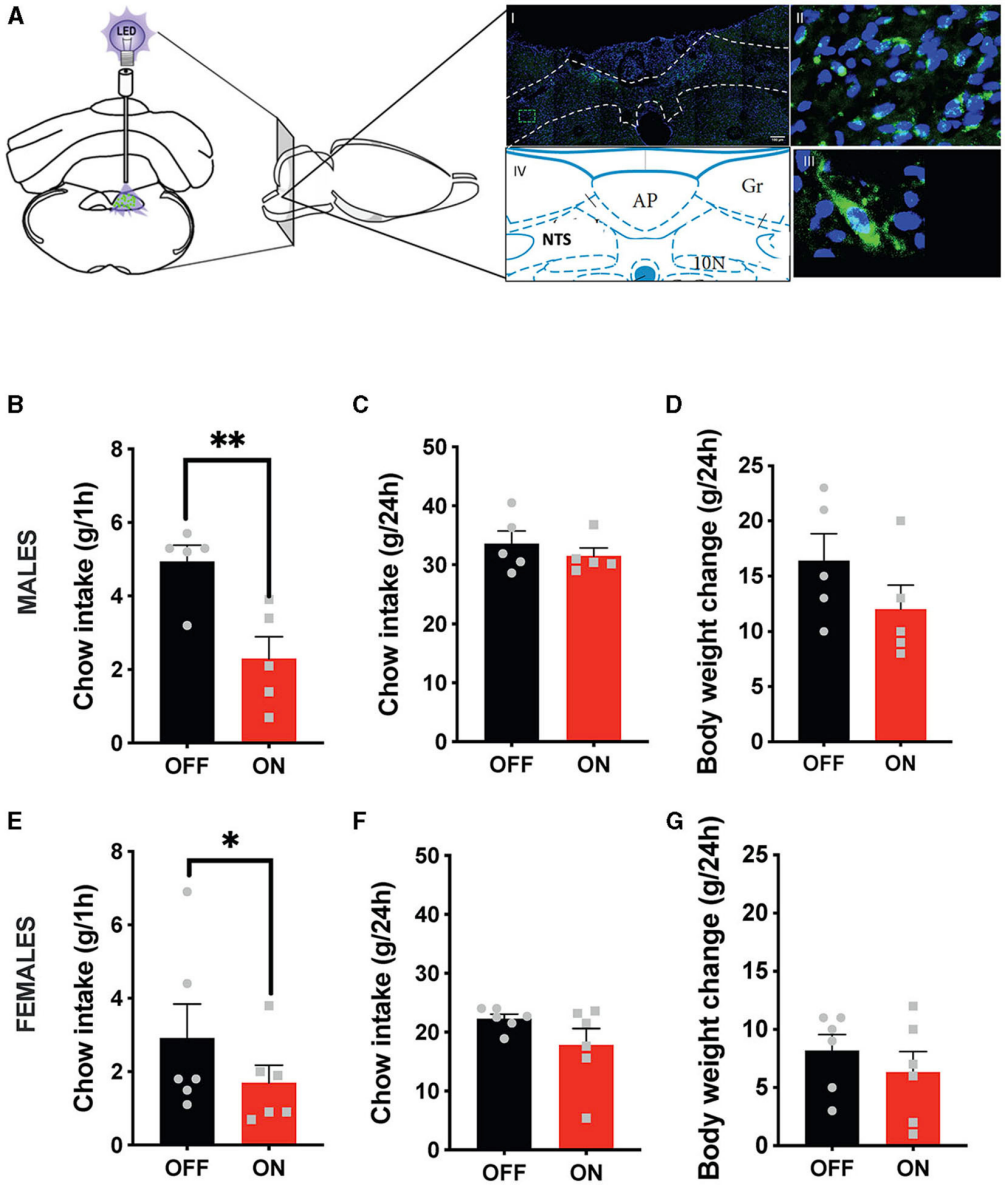
Anorexic effect of NTS GLP-1-producing neuron activation. Illustration of the optogenetic stimulation of the NTS *Gcg* neurons and a representative brain section showing expression of EGFP (green) in NTS *Gcg* cells in the Het *Gcg*-*Cre* rat **(A)**. Male rats reduce their 1 h chow intake in response to optogenetic activation of NTS *Gcg* neurons **(B)**. This anorexic response is subsided by the 24-h measurement time point **(C)**. No significant effect was found on the 24-h body weight change **(D)**. Female rats reduce their chow consumption at the 1-h measurement time point after optogenetic activation of NTS *Gcg* neurons **(E)**. There was no significant effect of the treatment on chow consumption at the 24-h measurement time point in female rats **(F)**. Body weight was also not altered in female rats **(G)**. Data are expressed as mean ±SEM. *n* = 5 for males and *n* = 6 for females. *P < 0.05, **P < 0.01.

Both sexes potently reduced their ingestive behavior after GLP-1-producing neuron optogenetic stimulation (Figure 1). Male rats consumed on average < 50% of their sham-stimulated chow intake 1 h after receiving food (Figure 1B). It is noteworthy that this anorexic effect was incredibly consistent, where 6 out of 6 tested males responded with reduced chow intake, and similarly, 5 out of 6 females. This effect dissipated by the 24-h measurement point (Figure 1C), and at that point, body weight was also not affected. Similarly, female rats consumed significantly less chow at the 1 h time point (Figure 1E) without any significant effect at 24 h on chow intake (Figure 1F) or body weight. A two-way ANOVA revealed a trend for interaction between treatment and sex (F (1, 9) = 4.265; *P* = 0.0689), a significant effect of treatment (F (1, 9) = 31.31; *P* = 0.0003), and no significant effect of sex (F (1, 9) = 2.224; *P* = 0.1700) on the 1 h food intake parameter. For 24-h food intake there was no significant interaction (F (1, 9) = 0.5010; *P* = 0.4970) and a borderline significant effect of treatment (F (1, 9) = 3.804; *P* = 0.0829), likely largely driven by female results (*P* = 0.12). There was also a significant effect of sex (F (1, 9) = 31.12; *P* = 0.0003). For body weight changes there was no significant interaction (F (1, 9) = 0.4277; P=0.5295) and no significant effect of treatment (F (1, 9) = 2.523; *P* = 0.1467), but there was a significant effect of sex (F (1, 9) = 13.60; *P* = 0.0050).

### Sex-divergent effect on food-motivated behavior of optogenetic stimulation of NTS *Gcg* neurons

**Figure 2:**
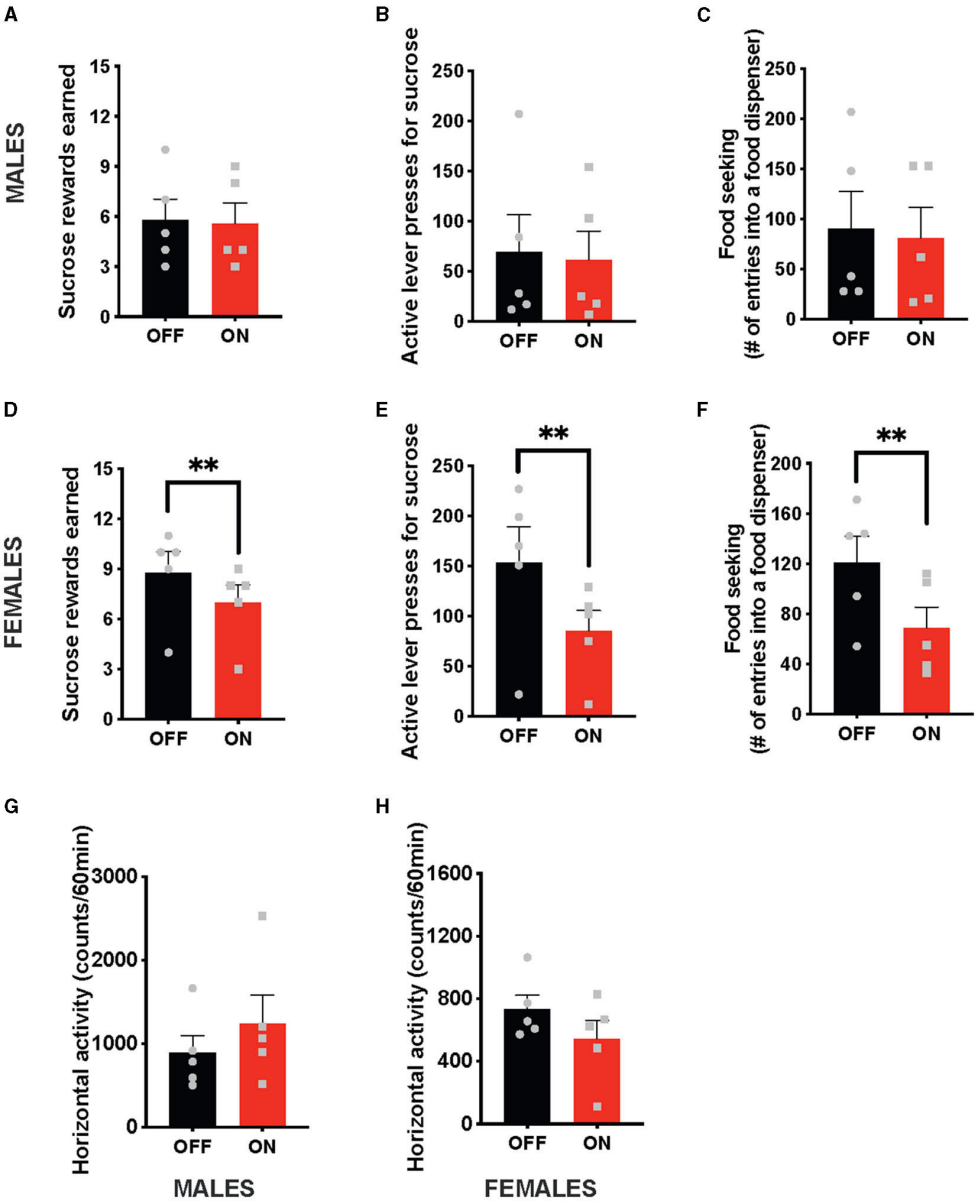
Sex-divergent effect on food-motivated behavior of optogenetic stimulation of NTS GLP-1-producing neurons. Photostimulation of the NTS *Gcg* neurons did not alter sucrose rewards earned **(A)** or lever presses for sucrose rewards **(B)** in male rats. Seeking food rewards was also not altered in males **(C)**. In contrast, the same photostimulation reduced the number of sucrose rewards earned **(D)** and the number of lever presses in female rats **(E)**. Female rats also displayed reduced food seeking after GLP-1-producing neuron photostimulation **(F)**. Locomotor activity was not affected by the optogenetic *Gcg* neuron activation in either sex **(G, H)**. Data are expressed as mean ±SEM. Gray squares show individual values. *n* = 5 for males and *n* = 5 for females. **P < 0.01.

In contrast to the anorexic effect obtained with the optogenetic stimulation of the NTS GLP-1-producing neurons, this treatment did not affect any parameters of food-motivated behavior in male rats (Figures 2A–C). Namely, the number of sucrose rewards earned (Figure 2A) and the number of lever presses males were willing to expend for the food reward (Figure 2B), or food seeking (Figure 2C) were not significantly different with or without the light on. In contrast, female rats reduced the number of sucrose rewards earned (Figure 2D) and the number of lever presses they were willing to expend for them (Figure 2E) after cNTS *Gcg* neuron activation. It is also noteworthy that this food-reward suppressing effect was incredibly consistent, where 100% of female rats tested reduced their food motivation. Optogenetically stimulated female rats also sought out the food rewards less compared to the sham stimulation condition (Figure 2F). Two-way ANOVA indicated a significant interaction between optogenetic activation and sex for the number of rewards earned (F (1, 8) = 9.14; *P* = 0.0165) as well as a significant effect of optogenetic stimulation on this parameter (F (1, 8) = 14.29; *P* = 0.0054). There was no significant effect of sex detected (F (1, 8) = 1.760; *P* = 0.2212). Similarly, a two-way ANOVA evaluating the number of active lever presses also indicated a significant interaction (F (1, 8) = 8.768; *P* = 0.0181), an effect of optogenetic stimulation (F (1, 8) = 14.22; *P* = 0.0055), and no effect of sex alone (F (1, 8) = 1.619; *P* = 0.2390). Furthermore, for food-seeking behavior, there was a significant interaction between sex and treatment (F (1, 8) = 5.778; *P* = 0.0429), a significant effect of treatment (F (1, 8) = 12.16; *P* = 0.0082), and no effect of sex alone (F (1, 8) = 0.05612; *P* = 0.8187). Neither male, nor female, rats altered their locomotion in response to the *Gcg* neuron photostimulation (Figures 2G, H), supported by two-way ANOVA results: treatment by sex (F (1, 8) = 1.453, *P* = 0.2625); treatment (F (1, 8) = 0.1257, *P* = 0.7321); sex (F (1, 8) = 4.560, *P* = 0.0652).

### Sex-divergent effect of chemogenetic activation of NTS *Gcg* neurons on food-motivated behavior

**Figure 3:**
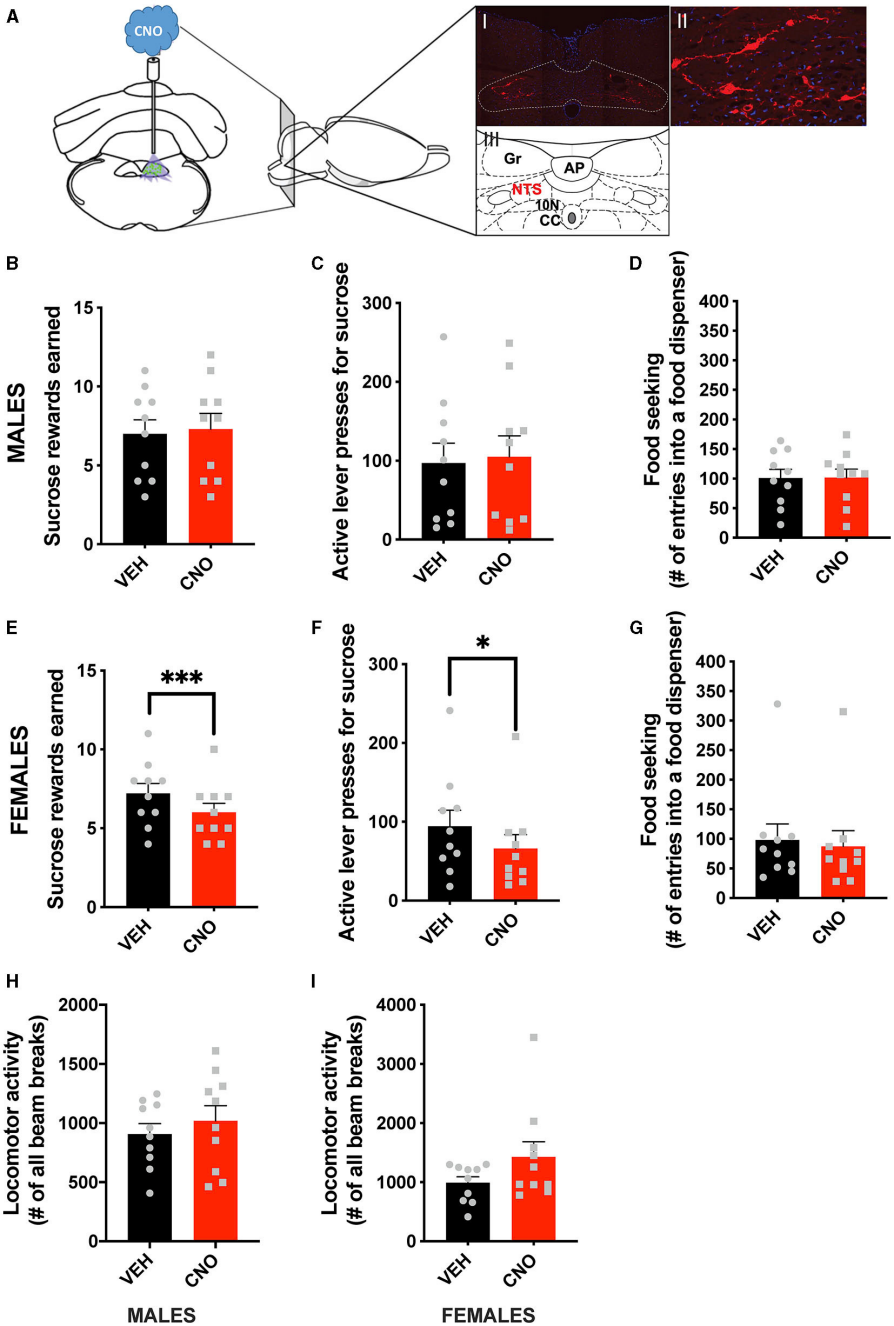
Sex-divergent effect of chemogenetic activation of NTS GLP-1-producing neurons on food-motivated behavior. Illustration of the chemogenetic activation of the NTS *Gcg* neurons and a representative brain section showing expression of mCherry (red) in NTS *Gcg* cells in the Het *Gcg*-*Cre* rat **(A)**. Chemogenetic activation of the NTS *Gcg* neurons did not alter sucrose rewards earned **(B)** or lever presses for sucrose rewards **(C)** in male rats. Seeking food rewards was also not altered in males **(D)**. In contrast, the same CNO injections reduced the number of sucrose rewards earned **(E)** and the number of lever presses **(F)**, but not food seeking **(G)** in female rats. Locomotor activity was not affected by the optogenetic *Gcg* neuron activation in either sex **(H, I)**. AP, area postrema; CC, central canal; 10 N, dorsal motor nucleus of vagus; Gr, gracile fasciculus. Data are expressed as mean ±SEM. *n* = 10 for males and *n* = 10 for females. *P < 0.05, ***P < 0.001.

In line with the results of the optogenetic stimulation, chemogenetic activation of NTS GLP-1-producing neurons did not affect food-motivated behavior in male rats (Figures 3A–D). The number of sucrose rewards earned (Figure 3B), lever presses emitted to obtain the sucrose rewards (Figure 3C), or food-seeking behavior (Figure 3D) were not altered by CNO injections in males. Importantly, and also in line with the optogenetic data, chemogenetic activation of NTS *Gcg* neurons resulted in the suppression of food-motivated behavior in females (Figures 3E–G). Both the number of sucrose rewards earned (Figure 3E) and the number of lever presses emitted for the rewards (Figure 3F) were reduced after CNO injections. Although, in contrast to the optogenetic results, food-seeking behavior remained unchanged (Figure 3G). Two-way ANOVA indicated a significant interaction between the chemogenetic activation and sex for the number of rewards earned (F (1, 18) = 14.78; *P* = 0.0012), as well as a significant effect of the chemogenetic activation on this parameter (F (1, 18) = 5.321; *P* = 0.0332), and no effect of sex alone (F (1, 18) = 0.2465; *P* = 0.6255). Similarly, two-way ANOVA evaluating the number of active lever presses also indicated a significant interaction (F (1, 18) = 6.160; *P* = 0.0232) between sex and treatment, no effect of treatment alone (F (1, 18) = 1.948; *P* = 0.1798) or sex (F (1, 18) = 0.4432; *P* = 0.5140). For food-seeking behavior, there was no significant interaction between sex and treatment (F (1, 18) = 0.9784; *P* = 0.3357), no effect of treatment (F (1, 18) = 0.7291; *P* = 0.4044), and no effect of sex (F (1, 18) = 0.08283; *P* = 0.7768). The treatment did not affect locomotor activity in either sex (Figures 3H, I); however, female rats tended to move more after the CNO injections (Figure 3I). No significant interaction was found for locomotor activity (F (1, 18) = 1.098; *P* = 0.3086), but a trend for an effect of CNO was revealed (F (1, 18) = 3.096; *P* = 0.0955), with no effect of sex alone (F (1, 18) = 2.324; *P* = 0.1448).

## Discussion

We demonstrate here that activation of NTS GLP-1-producing neurons in male and female rats leads to reduced ingestive behavior; thus, these neurons are sufficient to suppress feeding in rats of both sexes. Under the same activation conditions, food-motivated behavior was exclusively reduced in female rats, indicating that these neurons are sufficient to control food-motivated behavior in females but not males. Thus, collectively, we found there is a sex divergence in the ability of GLP-1-producing neuron activation to control motivated behavior for food.

Sex differences in the response to GLP-1R receptor activation are significant and distributed across the neuroaxis, but also very complex. We previously found that while systemic application of GLP-1R agonists to rats results in similar ingestive behavior suppression in both sexes, there is a more robustly motivated behavior suppression in females, compared to males, in certain physiological contexts (Richard et al., 2016). Yet, we also found a significantly less robust suppression of food-motivated behavior after pharmacological activation of GLP-1R in the lateral hypothalamus and the supramammillary nucleus, compared to male rats (Lopez-Ferreras et al., 2018, 2019). It is therefore conceivable that the lack of effect on this behavior in males after *Gcg* neuron activation acts as a buffer to the more sensitized response on the side of the GLP-1R. It may be that males depend predominantly on the very low levels of gut-produced GLP-1 that access the brain rather than the neuronally produced GLP-1 to suppress their motivated behavior via brain GLP-1R. However, we also found that in other brain areas, e.g., the ventral tegmental area, females show a stronger response to exogenous GLP-1R activation, in line with the overall stronger systemic response and the presence of motivated behavior suppression after *Gcg* neuron activation (Lopez-Ferreras et al., 2019). Ingestive behavior was similarly affected by pharmacological activation of GLP-1R in all brain areas probed in both sexes (Lopez-Ferreras et al., 2018, 2019). The important, and yet sparsely investigated, issue is whether the innervation of these different brain areas by *Gcg* neurons is overall similar among the GLP-1R-expressing and motivated behavior controlling brain areas in both sexes. It has already been shown that, surprisingly, not all physiologically relevant brain GLP-1R populations receive *Gcg* innervation (Hsu et al., 2015), at least in male rats. Moreover, even if the fibers are found in a given brain nucleus, it is still possible that GLP-1 is not released or is released only under restricted neuron activation conditions.

We have also previously found that estrogen enhances the effects of GLP-1 primarily on food-motivated behavior but also, to an extent, ingestive behavior (Richard et al., 2016, 2017; Vogel et al., 2016). Therefore, it would be tempting to hypothesize that in low estrogen cycle days (diestrus and metestrus), females may respond more like males and therefore not show reduced food motivation. However, considering that we see 100% of the females in our study responding with reduced motivated behavior after the optogenetic activation, the presence or absence of the effect is unlikely to be dictated by the estrous cycle, as we have measured estrous cycle in many previous studies and have never experienced a group that had exclusively been in estrus and proestrus at the same time on the same day. A possibility still remains, though, that the estrous cycle is associated with a more subtle and not all-or-none contribution, for example, a smaller contribution to the effect size.

The chemogenetic activation applied here was sufficient to release GLP-1 in males, as we have previously shown that the ingestive suppression after CNO injections in our *Gcg*-*Cre* rats is attenuated by a GLP-1R antagonist (Zheng et al., 2022). Furthermore, activated *Gcg* neurons produce more than just GLP-1; they have been shown to release glutamate and potentially also GLP-2, oxyntomodulin, and glicentin, among many other substances (Zheng et al., 2015b). It is possible that one or more of these substances synergize with GLP-1 to reduce food intake and motivated behavior. Whether *Gcg* neurons in female rats release the same co-transmitters as male *Gcg* neurons and in similar proportions is not yet known, as to date only male rats have been characterized. It is also conceivable that the action of endogenously released GLP-1 may synergize or be inhibited by the activity of other neurotransmitters, and this process may contribute to the lack of effect in male rats.

For our food-motivated behavior testing, we used a sucrose reward. Thus, an argument can be made that the sex-divergent results obtained here are restricted to sugar or carbohydrate-rich foods. However, our previous results with the same *Gcg*-*Cre* rat model indicate that, given a choice condition, palatable fat-rich food intake is reduced in females but not males after chemogenetic GLP-1-producing neuron activation (Zheng et al., 2022). This suggests that the identified sex divergence may not be limited to a specific food composition but instead spans both sugar- and fat-rich, palatable foods.

Food-motivated behavior may also be affected in males compared to females with different thresholds of GLP-1-producing neuron activation. The extent of *Gcg* neuron activation by the excitatory DREADD has been previously investigated in mice (Gaykema et al., 2017; Holt et al., 2019) and proven to be very extensive. While one study argued that chemogenetic activation of NTS GLP-1-producing neurons mimics a physiological signal, i.e., a normal-sized meal, a later study found that even a very large volume of a calorically dense solution did not manage to activate *Gcg* neurons to the extent achieved by CNO/hM3Dq (Holt et al., 2019). Even acute stress achieved a lesser activation than chemogenetic activation. Thus, given how powerful and efficient chemogenetic activation of *Gcg* neurons is, it is rather unlikely that a more powerful activation was needed for males or was physiologically or pathophysiologically relevant. Moreover, our conclusion that activation source or strength is not the driver of the sex divergence found here is further supported by two points. The first is the fact that an entirely different way of activating a neuron, namely photostimulation, also replicated the sex divergence. The second is that chemogenetic and optogenetic activation in males was effective at reducing ingestive behavior, indicating that activation was sufficient to alter behavior.

Brain GLP-1R and GLP-1-producing neurons affect behaviors and parameters outside of ingestive or motivated behavior, including anxiety-like behavior, body temperature, heart rate, and blood pressure (Hayes et al., 2008; Holt et al., 2020). Again, most of these studies have been done on male rodents or did not report the sex distribution of their subjects. Moreover, GLP-1R activation was shown to reduce motivated behavior (or intake) of various substances of abuse (Erreger et al., 2012; Egecioglu et al., 2013; Shirazi et al., 2013; Skibicka, 2013; Brunchmann et al., 2019; Douton et al., 2021). Thus, given our current results, a question ripe for investigation is whether the sex divergence in NTS GLP-1-producing neuron function in rats is uniquely relevant to food motivation or whether these results could be extended to substances of abuse.

The current study identifies a sex divergence in the role of GLP-1-producing neurons in the NTS in motivated behavior control. This finding paves the way for a myriad of important follow-up investigations evaluating the potential neuroanatomy and neurochemistry underlying this divergence and the role of gonadal hormones in these sex differences, as well as studies determining whether other parameters controlled by GLP-1R activation also differ between sexes and studies determining the role of the gonadal hormones in the discovered sex differences.

## Data availability statement

The original contributions presented in the study are included in the article/supplementary material, further inquiries can be directed to the corresponding author.

## Ethics statement

The animal study was approved by University of Gothenburg Animal Ethics Board. The study was conducted in accordance with the local legislation and institutional requirements.

## Author contributions

LL-F: Writing—review and editing, Data curation, Investigation, Formal analysis. MA: Writing—review and editing, Data curation, Investigation, Validation, Visualization. J-PK: Data curation, Investigation, Writing—review and editing, Visualization. KS: Writing—review and editing, Conceptualization, Funding acquisition, Resources, Supervision, Writing—original draft.
